# MHC-class-II are expressed in a subpopulation of human neural stem cells *in vitro* in an IFNγ–independent fashion and during development

**DOI:** 10.1038/srep24251

**Published:** 2016-04-15

**Authors:** B. Vagaska, S. E. P. New, C. Alvarez-Gonzalez, F. D’Acquisto, S. G. Gomez, N. W. Bulstrode, A. Madrigal, P. Ferretti

**Affiliations:** 1 Stem Cells and Regenerative Section, UCL Institute of Child Health, London WC1N 1EH, UK; 2 Anthony Nolan Research Institute, London NW3 2QG, UK; 3 UCL Cancer Institute, London WC1E 6DD, UK; 4 William Harvey Research Institute, Barts and The London School of Medicine, Queen Mary University of London, Charterhouse Square, London EC1M 6BQ, UK; 5 Anthony Nolan Cell Therapy Centre, Nottingham NG11 8NS, UK; 6 Department of Plastic Surgery, Great Ormond St. Hospital for Children NHS Trust, London WC1N 3JH, UK

## Abstract

Expression of major histocompatibility antigens class-2 (MHC-II) under non-inflammatory conditions is not usually associated with the nervous system. Comparative analysis of immunogenicity of human embryonic/fetal brain-derived neural stem cells (hNSCs) and human mesenchymal stem cells with neurogenic potential from umbilical cord (UC-MSCs) and paediatric adipose tissue (ADSCs), while highlighting differences in their immunogenicity, led us to discover subsets of neural cells co-expressing the neural marker SOX2 and MHC-II antigen *in vivo* during human CNS development. MHC-II proteins in hNSCs are functional, and differently regulated upon differentiation along different lineages. Mimicking an inflammatory response using the inflammatory cytokine IFNγ induced MHC-II up-regulation in both astrocytes and hNSCs, but not in UC-MSCs and ADSCs, either undifferentiated or differentiated, though IFNγ receptor expression was comparable. Together, hypoimmunogenicity of both UC-MSCs and ADSCs supports their suitability for allogeneic therapy, while significant immunogenicity of hNSCs and their progeny may at least in part underlie negative effects reported in some patients following embryonic neural cell grafts. Crucially, we show for the first time that MHC-II expression in developing human brains is not restricted to microglia as previously suggested, but is present in discrete subsets of neural progenitors and appears to be regulated independently of inflammatory stimuli.

The central nervous system (CNS) has been considered historically to be in an immunologically quiescent state^1^. This “immune privilege state” is due in part to the low expression of key regulators of the immune response, MHC class I (MHC-I) and class II (MHC-II) proteins, as well as the limited entry of infiltrating T cells into the CNS^1^,^2^. However despite this, induction of innate and adaptive immune responses occurs within the CNS following viral infection^1^. Furthermore, recognition of foreign MHC antigens on transplanted cells could be a crucial determinant for the immunological rejection of cell-derived products^2^,^3^.

Human neural stem cells (hNSCs) obtained from fetal tissue can successfully differentiate towards all different neural cell types^4^, and fetal cells are still considered the best option for neural cell therapy, as indicated by a recent decision of resuming clinical trials using such cells in patients with Parkinson’s disease^5^. In a human transplant paradigm, the fetal cell grafts have to be allogeneic, but the extent of immunoresponse they may elicit is still a matter of debate, as it is not possible to carry out these experiments in humans.

Different studies using *in vitro* models have suggested that allogeneic hNSCs and hNSCs derived from iPS^6^ or ES^7^ cells do not induce a significant immunoresponse. Odeberg *et al.* have suggested that although hNSCs express MHC, they are not immunogenic^8^. In contrast, potential hNSC immune response has been reported in other studies^9^,^10^. Also results from *in vivo* animal studies show discrepancy in their conclusions, with immunoresponse to neural stem cells reported to be low by some, and significant by others^11^,^12^,^13^.

The initial hypothesis we set to test was that expression of MHCs in hNSCs was comparable to that of mesenchymal stem cells (MSCs), that are considered to have low immunogenicity, though immuno-activation of these cells under inflammatory conditions has been suggested^14^,^15^,^16^, to have immunomodulatory properties, and to have the capacity to differentiate along the neural lineage^17^,^18^,^19^. We focused on mesenchymal cells that could be stably maintained and had the potential to be used for neural stem cell therapy, UC-MSCs (umbilical cord-derived MSCs) and paediatric ADSCs (adipose tissue-derived stem cells).

The finding that no MHC-II protein expression was observed in UC-MSCs and ADSCs, whereas a significant subset of hNSCs were positive, raised the issues of 1) the identity of these cells, as within the normal central nervous system (CNS) MHC-II are believed to be expressed only by microglia, and 2) their existence in the developing human CNS. We show here that the MHC-II-positive cells present in hNSC culture are not microglia as classified according to standard microglial markers, nor are simply an artifact of the *in vitro* system. As shown by analysis of MHC-II expression in hNSCs from different embryos, the MHC-II-positive population is constant through passages. Crucially, a subset of neural progenitors in the germinal zone, identified by SOX2 labeling, was found to co-express MHC-II in the embryonic human CNS. MHC-II in hNSCs are functional in recognizing allogeneic T cell receptors, and, unlike ADSCs, are rapidly killed by T cells. MHC-II expression *in vitro* does not appear to be regulated via an autocrine mechanism, and all hNSC cells appear to have the potential to express MHC-II in response to IFN-γ stimulation. Finally, we show different regulation of MHC-II in hNSCs induced to differentiate along the astrocytic or neurogenic lineages, with down-regulation in the former and up-regulation in the latter. Together, our studies suggest the existence of a novel neural stem cell population within the developing human CNS constitutively expressing MHC-II, rather than as a consequence of an inflammatory reaction. Furthermore, these findings suggest that hNSCs and their progeny may mount an immune response when grafted for therapeutic purposes which may have serious implications for the outcome of the procedure and possibly account for negative outcome in some patients in clinical trials.

## Results

### MHC class I and II expression in hNSCs, ADSCs, and UC-MSCs

**Figure 1:**
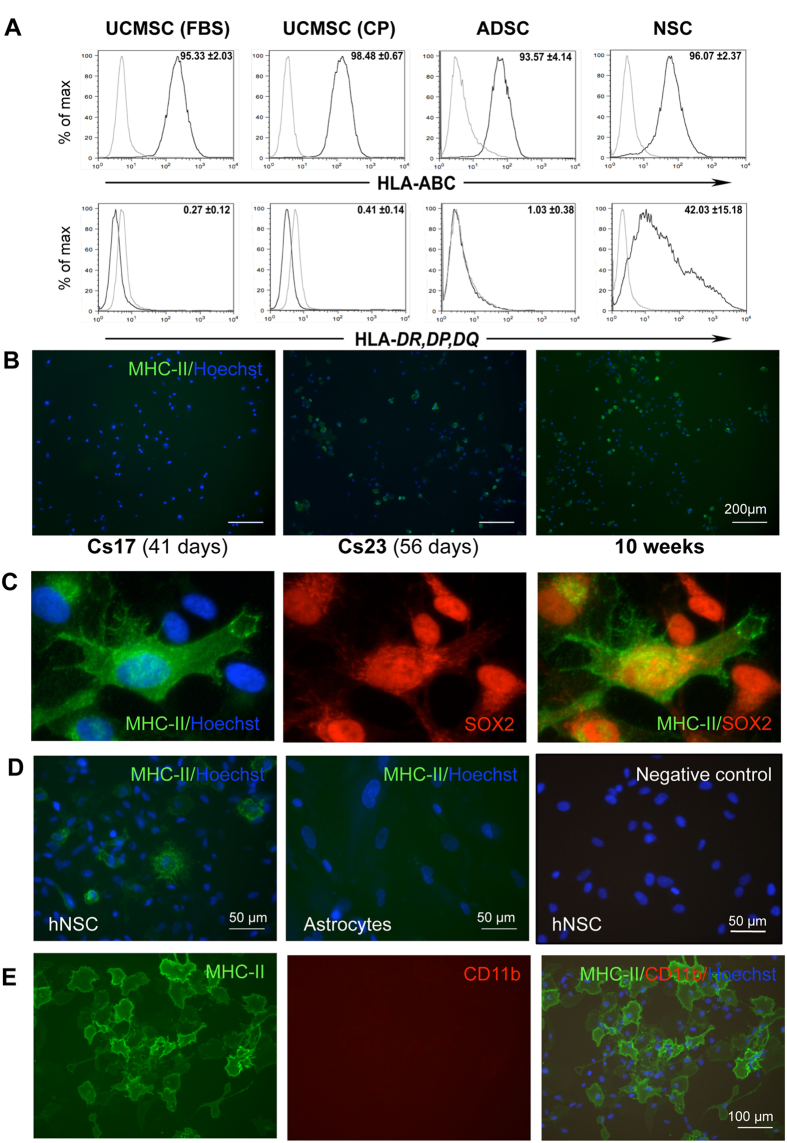
Detection of MHC proteins by flow cytometry and immunofluorescence staining of MHC class II (MHC-II) in combination with the neural stem cell marker, SOX2, and the microglial markers CD11b. (**A**) HLA-A, -B, -C (MHC-class I) and HLA-DR, -DP, -DQ (MHC-class II) assessed by flow cytometry in UC-MSCs (FCS), UC-MSCs (CP), ADSCs and hNSCs (light grey curves: negative controls; dark grey curves: labelled cells). These are representative histograms of ≥3 cell isolates. Data are percentages represented as mean ± SEM. Abbreviations: ADSC, adipose-derived stem cells; CP, cord plasma; FCS, fetal calf serum; HLA, human leukocyte antigens; NSC, neural stem cells; UCMSC, umbilical cord mesenchymal stem cell. (**B**) MHC-II staining in hNSCs from 3 brains at different stages of development. Cs: Carnegie stage. Nuclei are counterstained with Hoechst dye (blue). Note that subsets of hNSCs are positive at all stages. (**C**) hNSCs (Cs23) double-labelled for SOX2 and MHC-II. All nuclei are SOX2-positive, and some cells display also cytoplasmic SOX2 staining; a subset of SOX2-positive cells expresses MHC-II protein. (**D**) hNSCs and astrocytes stained for MHC-II; positive cells are observed in hNSCs but not in astrocyte cultures. (**E**) hNSCs stained for MHC-II and CD11b; no CD11b-positive cells are observed.

Potential immunogenicity of hNSCs, UC-MSCs and ADSCs was first investigated by comparing expression of the major histocompatibility complexes, HLA-A, -B, -C (MHC class I, MHC-I) and HLA-DR, -DP, -DQ (MHC class II, MHC-II), by flow cytometry analysis in 3 independent lines for each cell type. MHC-I was detected in the majority of hNSCs, UC-MSCs and ADSCs (Fig. 1A). UC-MSCs and ADSCs were negative for MHC-II on a basal level whilst approximately 50% (average from 3 different hNSC lines) of undifferentiated hNSCs expressed MHC-II (Fig. 1A). Immunofluorescence staining of the 3 hNSC lines confirmed expression of MHC-II and showed that MHC-II-positive cells were also positive for neural stem cell markers, such as SOX2 (Fig. 1B,C), nestin, PAX6 and Tbr2 (Supplementary Fig. S1A–C). The difference in the proportion of MHC-II-positive cells in each line did not appear to change through passages. In contrast to hNSCs, human astrocytes were negative for MHC-II (Fig. 1D).

The hNSC lines used here and by others obtained with the same protocol have been thoroughly characterized as SOX2-positive neural stem cells^4^ and we have previously shown by FACS analysis that ≥98% of the hNSCs are SOX2-positive^20^. Nonetheless, to rule out that the MHC-II staining observed might be due to microglia contaminations, we stained our cultures with microglial markers, such as CD68 and CD11b. hNSCs were completely negative for CD11b (Fig. 1E) and CD68 (Supplementary Fig. S1D).

### MHC class II expression in the embryonic human nervous system

**Figure 2:**
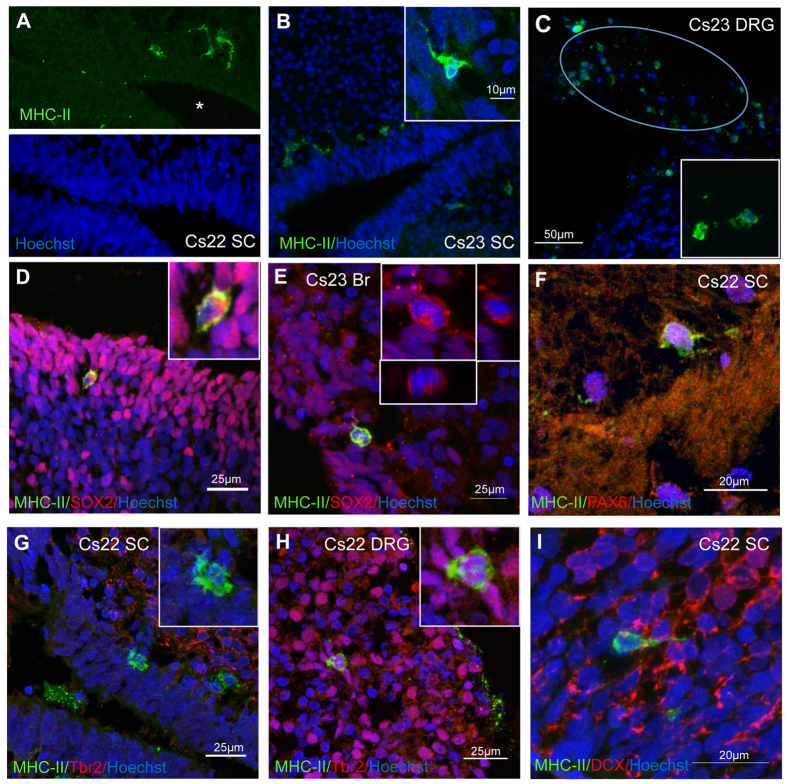
Detection of MHC class II and neural markers in human fetal CNS by immunofluorescence. (**A–C)** MHC-II staining in spinal cords (SC) and dorsal root ganglion (DRG) from developing embryos at Carnegie stages 22 (Cs22) and 23 (Cs23). Note expression of MHC-II protein in a subset of cells in the germinal layer surrounding the central canal. The dorsal root ganglion, indicated by the oval in (**C**) contains several MHC-II-positive cells. The inserts show a group of positive cells at a higher magnification. (**D,E)** Double-staining for MHC-II and SOX2 in Cs23 spinal cords (**D**) and brain (**E**). MHC-II staining is detected in cells expressing SOX2 mainly in the cytoplasm (insert). (**F**) Spinal cord stained for PAX6 and MHC-II at higher magnification. PAX6 protein expression is detected in MHC-II positive cells. (**G,H**) Double-staining for Tbr2 and MHC-II protein expression in spinal cord (**G**) and DRG (**H**); note the absence of co-labelling in spinal cord cells, but co-expression in MHC-II positive cells in DRG. (**I**) Cs22 spinal cord stained for MHC-II and doublecortin (DCX) proteins; no double-labelled cell is detected.

We then investigated whether a population of neural stem cells that expresses MHC-II exist *in vivo* by immunostaining brain and spinal cord sections from human embryos. A small number of MHC-II-positive cells per section (n ≥ 2) was found in all sections (n ≥ 6/embryo) stained from 5 different embryos at Carnegie stages 22 (Cs22) and 23 (Cs23); these cells were located in the proximity or within the periventricular zone of the spinal cord and brain where the SOX2-positive neural progenitors reside (Fig. 2A,B). A subset of MHC-II-positive cells was present also in the dorsal root ganglia, within a population defined in other studies as “support cells” (Fig. 2C).

Double-labelling for MHC-II and SOX2 showed that most of the MHC-II-positive cells were also SOX2-positive (>87%), consistent with the co-expression of these proteins observed *in vitro* (Fig. 2D,E and Supplementary Table 1). Co-staining of spinal cord sections with additional neural markers, showed that subsets of MHC-II positive cells expressed PAX6 (Fig. 2F) and nestin (Supplementary Fig. S3). At the developmental stage examined (Carnegie stage 22), co-expression of MHC-II and Tbr2, that in the mouse is up-regulated during the transition from radial glia to intermediate progenitor cells^21^ and is expressed in hNSCs *in vitro* (Supplementary Fig. S1C), was detected in the dorsal root ganglion but not in the spinal cord (Fig. 2G,H). Whereas cells co-expressing MHC-II and neural stem cell markers were consistently detected (≥2 double-labelled cells/section), no cell double-labelled for MHC-II and the neuroblast marker, doublecortin, was observed (Fig. 2I).

**Figure 3:**
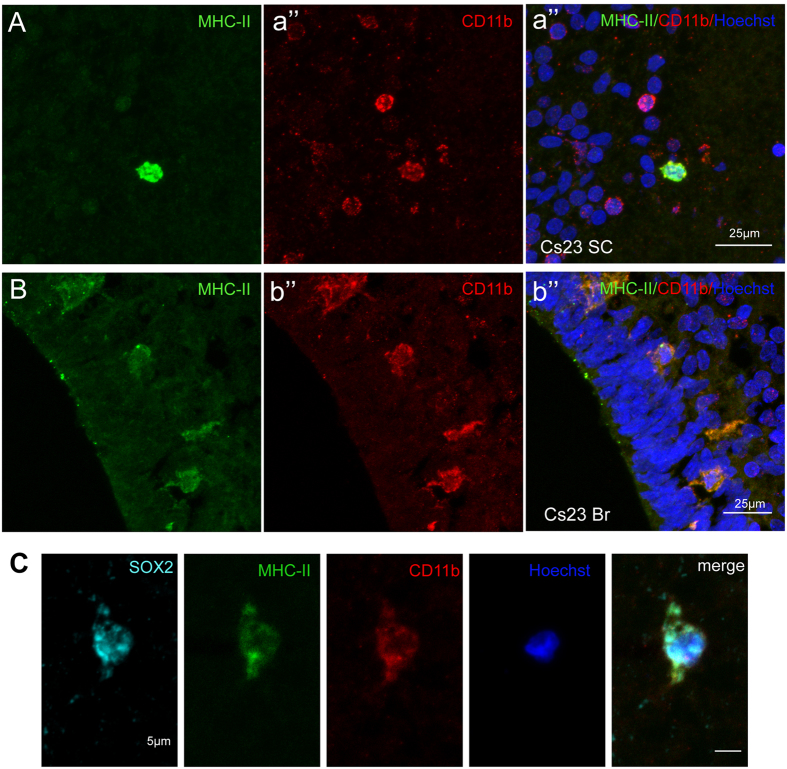
MHC class II (MHC-II), CD11b and SOX2 expression in human fetal CNS detected by immunofluorescence. (**A,B**) Double-staining for MHC-II and CD11b in spinal cord (SC) and brain (Br) from embryos at Carnegie stages 23 (Cs23). Note that not all CD11b positive cells are MHC-II positive. (**C**) Triple staining for MHC-II, CD11b and SOX2 in developing spinal cord at Cs23 shows presence of a subset of cells positive for all three markers.

To further investigate the identity of MHC-II-positive-cells *in vivo*, spinal cord and brain sections were double stained for MHC-II and the microglial markers, CD11b (Fig. 3) and CD68 (Supplementary Fig. S4). As exemplified in Fig. 3A,B, cells expressing CD11b were found both in brain and spinal cord in Cs23 embryos, and more than 90% of the CD11b-positive cells was also MHC-II positive. Finally, triple staining for MHC-II, CD11b and SOX2 in developing spinal cord was carried out at Cs23 (Fig. 3C) and demonstrated the presence of a subset of cells positive for all three markers (>92% of MHC-II-positive cells, Supplementary Table S1). This indicates that the human developing nervous system contains a sub-population of SOX2-positive cells that expresses also MHC-II and CD11b.

### Functional analysis of MHC-II

**Figure 4:**
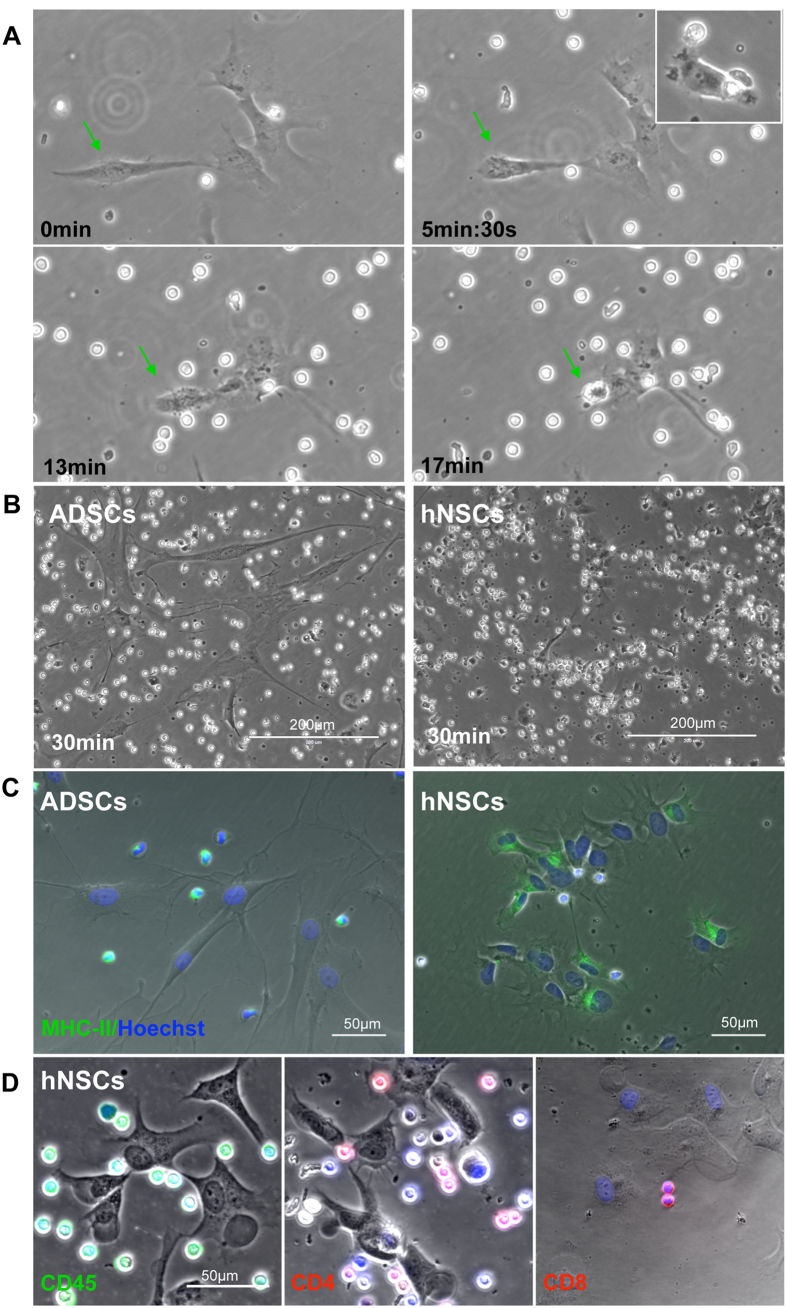
Response of hNSCs and ADSCs to T cell detected by time-lapse photography and immunofluorescence. (**A**) Frames taken at different times of co-culture of hNSCs and T cells. (**B**) Comparison of hNSCs and ADSCs response to T cells after 30 minutes in co-culture. (**C**) MHC-II staining of hNSCs and ADSCs co-cultured with T cells. MHC-II reactivity is observed in hNSCs and T cells, but not in ADSCs. (**D**) Expression of CD45, CD4 and CD8 in hNSC and T cell co-cultures; note that all blood-derived cells are CD45-positive and that both CD4- and CD8-positive T cells make contact with hNSCs.

In order to establish whether MHC-II proteins expressed by hNSCs are functional, we carried out a T cell co-culture assay using allogeneic T cells from a donor of different sex from the hNSCs. As shown in Fig. 4A (frames from time-lapse imaging) and in Movies 1–2, T cells rapidly formed synapses with the hNSCs. Within a few minutes from adding the T cells, significant morphological changes were observed: 30.9% ± 5.3 of hNSCs had started to shrink, as evaluated from images collected at 30 minutes of co-culture, and then died (Fig. 4A,B). In contrast, ADSCs did not evoke a T cell response (Movie 3), consistent with their lack of MHC-II expression, and neither morphology nor their survival were affected (Fig. 4C). Immunostaining of co-cultured cells for the T cell markers, CD4 and CD8, showed that both CD4 and CD8 positive T cells interact with hNSCs (Fig. 4D).

**Figure 5:**
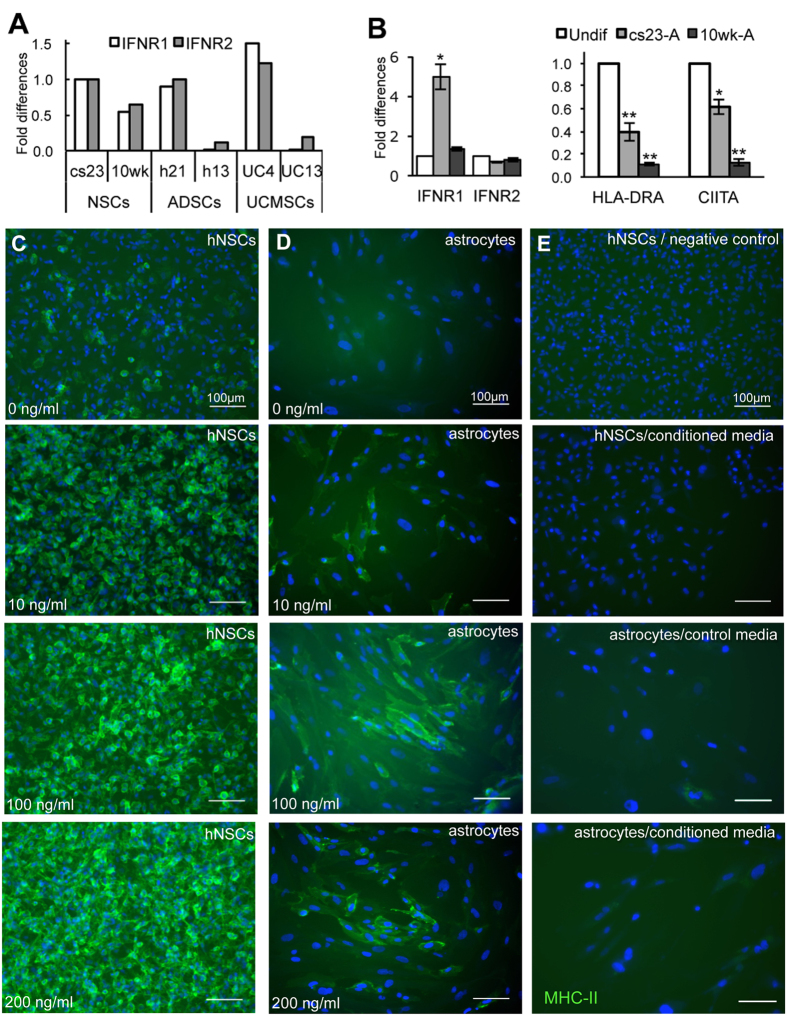
Expression of IFNγ receptors (IFNR1 and IFNR2) and effect of IFNγ stimulation on MHC class II (MHC-II) expression in undifferentiated hNSCs (cs23) and astrocytes differentiated from the same line. (**A**) Expression of IFNR1 and IFNR2 in hNSCs, ADSCs and UC-MSCs assessed by RT-qPCR. (**B**) Fold changes in the expression of IFNR1 and IFNR2, and HLA-DRA and CIITA in astrocytes in relation to their parental hNSCs prior to differentiation assessed by RT-qPCR (Mean ± S.E.M; n = 3, biological replicates); *p < 0.05; **p < 0.01 (Student’s t-test). (**C**) Effect of IFNγ stimulation on hNSCs. Note the dose-dependent increase of MHC-II reactivity in hNSCs. (**D**) Effect of IFNγ stimulation on astrocyte; note MHC-II up-regulation upon IFNγ-treatment. (**E**) Negative control of the MHC-II staining (top panel) and stimulation of astrocytes or hNSCs with low MHC-II expression with hNSC conditioned medium (below); no MHC-II induction is observed. All scale bars are 100 μm.

We then assessed expression of the IFNγ receptors, IFNγR1 and IFNγR2, in hNSCs, UC-MSCs, ADSCs and astrocytes by RT-qPCR to establish whether these cells had the potential to respond to IFNγ stimulation. As shown in Fig. 5A,B, all cell types expressed both IFNγ receptors. However, the mRNA levels of HLA-DRA and CIITA (class II transactivator), the master regulator that modulates both constitutive and IFNγ-induced MHC-II expression, were significantly reduced in astrocytes as compared to their parental cell lines (Fig. 5B). Having observed that a subset of hNSCs expressed MHC-II proteins, but astrocytes, UC-MSCs and ADSCs did not (Fig. 1), we investigated whether MHC-II was up-regulated in the three hNSC lines upon stimulation with IFNγ. MHC-II expression in hNSCs was clearly increased by treatment with 10 ng/ml, with virtually all cells becoming positive when treated with 200 ng/ml IFNγ (Fig. 5C). Dose-dependent induction of MHC-II by IFNγ was observed also in astrocytes; the apparently less intense staining is lilkely due to the flatter morphology of astrocytes as compared to hNSCs (Fig. 5D). In contrast, no MHC-II reactivity was observed in either UC-MSCs or ADSCs stimulated with IFNγ, nor following their differentiation (Supplementary Table S2, Supplementary Fig. S5A–J), independently from the levels of IFNγ receptor expression that were largely comparable to those detected in hNSCs (Fig. 5A, Table 1). These results show that whereas hNSCs increased MHC-II expression in response to IFNγ, undifferentiated UC-MSCs or ADSCs did not.

We wished to establish whether expression of MHC-II in hNSCs could be due to autocrine effects. Hence we first assessed whether these cells expressed IFNγ. As we could not detect any significant amount of IFNγ transcript either by RT-PCR or by RT-qPCR, we hypothesised that other components secreted by hNSCs might induce MHC-II expression. To this purpose we tested the effect of hNSC conditioned medium on MHC-II expression in the MHC-II –negative astrocytes and in hNSCs lines with low levels of MHC-II expression. The conditioned medium did not induce MHC-II expression in either of them suggesting that regulation of their expression in hNSCs is not via an IFNγ-dependent autocrine mechanisms (Fig. 5E).

### Expression of MHC-II following hNSC differentiation

**Figure 6:**
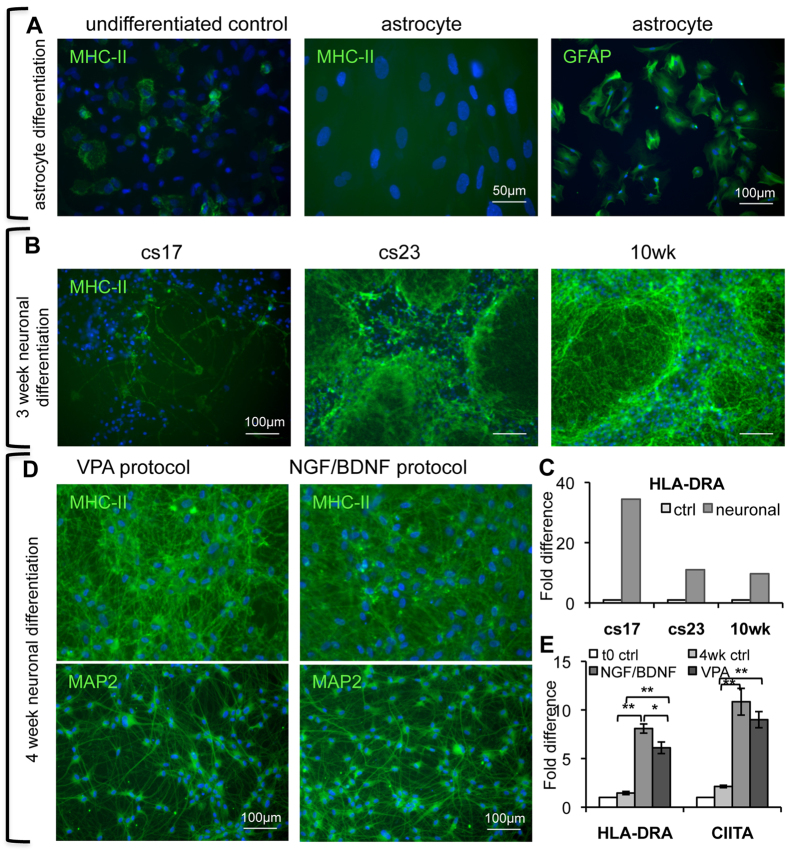
MHC class II (MHC-II) expression in undifferentiated hNSCs and following neuronal and astrocytic differentiation. (**A**) Detection of MHC-II protein by immunocytochemistry in undifferentiated hNSCs and following astrocytic differentiation: differentiated astrocytes are MHC-II-negative and GFAP-positive. (**B**) Detection of MHC-II protein by immunocytochemistry in hNSCs from 3 different developing brains following neuronal differentiation for 3 weeks. Note the high expression of MHC-II protein in subsets of differentiated cells and increased expression upon differentiation. (**C**) Detection of MHC-II mRNA by RT-qPCR (values are means of 2 biological replicates) in the hNSC lines shown in (**B**); note that the MHC-II transcript increases in all lines upon differentiation. (**D**) Detection of MHC-II proteins by immunocytochemistry following differentiation of hNSCs (cs23) for 4 weeks using two different protocols; increased MHC-II expression is observed in both conditions. (**E**) Detection of HLA-DRA and CIITA mRNAs by RT-qPCR in hNSCs (cs23) under the differentiation conditions shown in (**B**). Mean ± S.E.M (n = 3, biological replicates; *p < 0.05; **p < 0.001 (one-way ANOVA followed by Tukey t-test).

We compared regulation of MHC-II expression following hNSC differentiation along the astrocytic and neurogenic lineages to establish whether MHC-II down-regulation was a general feature of hNSC differentiation. hNSCs induced to undergo astrocytic differentiation were GFAP-positive, as expected for astrocytes, and did not show any MHC-II reactivity (Figs 1C and 6A). Induction of neuronal differentiation of hNSCs resulted in morphological changes and up-regulation of MAP2 protein, as well as in an increase in both MHC-II protein and transcript in all lines (Fig. 6B,C). In addition, CIITA expression was significantly increased in the differentiated cultures (Fig. 6E). Valproic acid (VPA), one of the differentiation medium components, is a histone deacetylase inhibitor, and this class of compounds has been shown to regulate CIITA and MHC-II expression^22^,^23^,^24^. Hence, we assessed whether VPA affected MHC-II expression in somatic stem cells; no obvious change was observed either in hNSCs or ADSCs (Supplementary Fig. S6). Furthermore, we examined MHC-II reactivity following hNSC neuronal differentiation using a growth factor (NGF/BDNF) protocol, in which the culture medium had a completely different composition. MHC-II up-regulation was observed also under these experimental conditions (Fig. 6D,E). MHC-II expression increase after neuronal differentiation was comparable in the two protocols, both at the protein and RNA level. This suggests that the increase in MHC-II is regulated independently from the composition of the culture medium.

We further assessed MHC-II expression in control and differentiated ADSC and UC-MSCs (Supplementary Figs S5–7 and Supplementary Table S2). Neither of these stem cell populations expressed MHC-II, though a few positive cells could be detected in sections of fat tissue. Up-regulation of MHC-II in these cells did not occur either following neuronal differentiation or differentiation along mesenchymal lineages, and the levels of MHC-II and CIITA transcripts in both undifferentiated and neurally differentiated hADSCs were much lower than those detected in hNSC (Table 1, Supplementary Fig. S7A,B). Finally, as differentiated UC-MSCs and ADSCs expressed both CIITA and IFNγ receptors (Table 1, Supplementary Fig. S7), we assessed whether upon differentiation these cells would respond to IFNγ by increasing MHC-II expression. No MHC-II reactivity was detected in the differentiated IFNγ-treated cells by immunocytochemistry (Supplementary Table S2).

## Discussion

In this study we provide evidence for a novel population of MHC class II-expressing progenitors that reside in the developing human CNS. Our results suggest that these MHC-II molecules are not only functional, but also that they are regulated in an IFNγ-independent fashion. Importantly, these findings question the widely accepted notion that microglia are the only MHC class-II expressing cells in the central nervous system. To our knowledge this is the first time the existence of a population of MHC-II/SOX2-positve cells in the germinal zone of the human embryo has been shown. Furthermore, the potential high immunogenicity of hNSCs is demonstrated in parallel with the hypo-immunogenicity of the mesenchymal UC-MSCs and ADSCs. This may have important implications for the brain immune response to cell or embryonic neural tissue grafting.

### MHC-II reactivity in neural stem cells identify a novel cell population in the developing human CNS

Our results show that MHC-II molecules are expressed in neural progenitors both *in vitro* and *in vivo*. We could not find any evidence based either on morphology or expression of microglial markers in our hNSC cultures that would indicate that microglial contamination account for the significant expression of MHC-II we observed; in addition, the proportion of MHC-II positive cells did not obviously change with passaging. This supports the view that MHC-II-positive hNSCs represent a distinct proliferating subpopulation of neural progenitors and the findings *in vivo* are consistent with this hypothesis.

Together our results support the hypothesis of a constitutive rather than autocrine regulation of MHC-II expression in hNSCs. IFNγ treatment increased expression in hNSC in a dose-dependent manner, and, importantly, showed that all hNSCs have the potential of expressing MHC-II. Furthermore, IFNγ mRNA was hardly detectable in hNSCs and hNSC-conditioned medium could not elicit an immune response in astrocytes, unlike treatment with IFNγ. Finally, MHC-II expression in SOX2-positive cells was consistently found also in the developing human CNS at stages when the immune system is still poorly developed. Hence, it is most unlikely that infective agents had triggered an immune response within the embryonic CNS in all these pregnancies.

An apparent difference in the MHC-II expressing SOX2 positive neural progenitors observed *in vivo* is their co-expression of microglial antigens, CD11 and CD68. These findings open questions about the origin and identity of these cells in the developing human CNS. Unfortunately, as tracking studies are not feasible in humans, at this stage we can only speculate. It is well established that the SOX2-positive hNSCs cells have neuronal and glial differentiation potential. The fact that some hNSCs co-express MHC-II and SOX2 suggests a neuroectodermal origin of these cells. However, an alternative intriguing possibility is that, as proposed in a study carried out in rats, microglia harbour a neuronal and glial differentiation potential that is more readily expressed under culture conditions^25^. Whether such a microglial population really exists *in vivo* and might be of neuroectodermal rather than of mesodermal origin has yet to be seen. Currently we cannot rule out the possibility that old suggestions of a dual origin of human microglia based on animal work might have been prematurely dismissed^26^,^27^,^28^.

We have also shown a great up-regulation in MHC-II upon hNSCs neuronal differentiation. Interestingly, recent studies have suggested a role for MHC-I in neurite outgrowth and synaptic plasticity in the developing brain, and that dopaminergic neurons *in vitro* are particularly prone to up-regulate MHC-I in response to inflammatory stimuli^29^,^30^,^31^,^32^,^33^,^34^. It will be important to establish whether MHC-II expression in human neurons *in vitro* reflects transient *in vivo* developmental events and whether it is restricted to specific neuronal cell types and plays specific roles in neural development. For example, given the proposed cross-talk between neurons and microglia in the normal brain, transient MHC-II expression in embryonic neurons might play a role in microglia development^35^. Based on the differences in MHC-II expression observed in neurons *in vivo* and *in vitro* it is conceivable that interactions between neurons with other cell types negatively regulate expression of MHC-II in the developing and possibly in the adult brain. Hence dys-regulation of these interactions may contribute to disease states such as in neurodegenerative diseases. There is some indication that this is the case for MHC-I, whose expression renders neurons particularly susceptible to T-cell mediated degeneration^34^. The scarcity of relevant human tissue and the possibility of manipulating it is a limitation to providing rapid answers to these important questions.

### MHC-II, but not MHC-I, is differentially expressed in neural and mesenchymal stem cells and associated with high immunogenicity

Our comparative analysis of immunogenicity of hNSCs and mesenchymal stem cells, paediatric ADSCs and UC-MSCs, highlights important differences among these cell lines. In all the cell lines studied most cells express MHC-I. In contrast, only hNSCs, which are rapidly killed by T cells, express MHC-II at significant levels. MSCs, either undifferentiated or differentiated, do not express MHC-II, nor respond to IFNγ or interact with T cells, consistent with a previous report^36^. This supports the view that MHC-II is the main antigenic complex underlying differences in the immunogenicity of these cells.

The immunogenicity of hNSCs derived from human embryos/fetuses between 41 days and 10 weeks of gestation is more extensive than previously reported and it dramatically increases upon neuronal differentiation. The increase in MHC-II expression in hNSCs differentiated towards the neuronal lineage was unexpected. In a previous study human neural progenitors grown as neurospheres did not up-regulate MHC-II upon neuronal differentiation^10^. MHC-II up-regulation in our cultures was neuron-specific; it was observed with two completely different neuronal differentiation protocols, and did not occur upon differentiation towards the astrocytic lineage, when, in contrast, MHC-II was down-regulated. Nonetheless, the astrocytes expressed the IFNγ-receptors, and up-regulated MHC-II expression in an IFNγ-dependent pathway, demonstrating the potential for mounting an immune response in an inflammatory environment.

Our hNSCs differed from those described by Odeberg *et al.* and Laguna Goya *et al.* also in regard to their behaviour in co-cultures with T cells^8^,^10^. Whereas these authors could co-culture their neural progenitors with T cells for several days to assess their ability to induce T cell proliferation, our hNSCs derived from 3 different embryos died when exposed to allogeneic T cells, and particularly rapidly when sex-mismatched. In our study we used post-adherent T lymphocytes (>98% pure) rather than the whole population of PBMCs as used in the study by Odeberg, or purified CD4+ or CD8+ T-cells as in the Laguna Goya’s study, where 6.5% of neural stem cells were MHC-II-positive^8^,^10^. It should be noted that the CD4+ and CD8+ T-cells increased their proliferative activity when co-cultured with neural stem cells suggesting that an immunogenic response had been triggered. Another difference between ours and other studies is in the neural stem/progenitor cells themselves, as we used different isolation and growth protocols. In both studies mentioned above, cells were expanded as neurospheres, while our hNSCs are grown on laminin as a monolayer, which has been previously reported as the preferable method of culture to maintain the phenotype of stem cells stable over time in culture^37^. It is therefore possible that the differences between the studies reflect differences in the populations of neural stem cells isolated and expanded *in vitro*. While expression of MHC-II in neural stem cells in the embryo is likely to play a physiological role, it is apparent that they could mount an immune response upon transplantation. Notwithstanding some differences in the results between our study and the one by Laguna Goya, there is indeed agreement on the key issue that much caution is required in the use of hNSCs and their progeny in cell therapies. MHC-II compatibility and use of immunosuppression should be considered to maximize the chances of survival and successful engraftment of undifferentiated or differentiated (e.g. dopaminergic neurones) hNSCs.

In contrast to hNSCs, none of the ADSC and UC-MSC lines seem to be immunogenic even when induced to express neuronal and glial markers, though less efficiently than hNSCs^38^. Although it might prove difficult to differentiate them into specific mature neuronal sub-types, they can play important trophic and/or immunomodulatory roles in injured CNS^39^,^40^. This, together with their low immunogenicity, allowing them to survive longer than hNSCs after grafting as reported in animal models^41^, makes ADSCs and UC-MSCs attractive cell sources for aiding repair after traumatic brain injury or hypoxia-ischaemia.

## Conclusions

We have identified a previously undiscovered population of MHC-II/SOX2-positive cells in the germinal zone of the human embryo that does not appear to be due to an inflammatory response. MHC-II expression in human neural progenitors is found both *in vitro* and *in vivo*, and also in neurons at least *in vitro*. This is consistent with a non-immune role of MHC-II in the human developing CNS, as it occurs before maturation of the adaptive immune system, but might play a role in its development.

Furthermore, the fact that hNSCs can evoke a robust immune response may have important implications for the use of embryonic/fetal neural cells therapeutically and perhaps account, at least in part, for unexplained poor outcomes noted in some patients during clinical trials utilizing neural cell therapies.

## Methods

All chemicals were from Sigma-Aldrich (UK), unless otherwise stated.

All procedures involving human tissue were carried out in accordance to the UK Human Tissue Act 2006.

### Human embryonic and fetal tissues

Hindbrains and spinal cords from human embryos between 6–10 weeks of gestation from consenting patients were provided by a tissue bank, the Human Developmental Biology Resource (HDBR, http://hdbr.org) under ethical approval (NRES Committee London – Fulham, UK). Dissected tissues were either dissociated for culturing as described below, or fixed in 4% PFA prior to cryo-embedding and sectioning for protein expression analysis by immunofluorescence. Embryos at Carnegie stages 22 (approximately 53 days of gestation) and 23 (approximately 56 days of gestation) and a fetus at 10 weeks of gestation were used in this study.

### Cell growth, differentiation and treatments

All cells were grown at 37 °C with 5% CO_2_ in a humidified incubator.

#### Human Neural Stem Cells (hNSCs)

hNSCs were isolated as previously described^4^,^42^. Cells were grown in DMEM/F12 medium with Glutamax (Life Technologies) containing 1% penicillin/streptomycin (Life Technologies), 1% N2 supplement, 2% B27 supplement (both Life Technologies), 20 ng/ml human recombinant fibroblast growth factor 2 (FGF2), 20 ng/ml human recombinant epidermal growth factor (EGF; both Peprotech), 50 μg/ml bovine serum albumin (BSA) fraction V and 5 μg/ml heparin, and laminin was added to the medium (10 μg/ml) instead of coating the dishes. Most experiments were carried out with cell at passage 10–20, in some experiments early (10) and late (30) passages were compared.

#### Human Pediatric Adipose tissue-Derived Stem Cells (ADSCs)

Abdominal adipose tissue was collected from consenting patients under ethical approval from the Camden and Islington Community Local Research Ethics Committee (London, UK). ADSCs were isolated from lipoaspirates of pediatric patients as previously described^17^. Isolated ADSCs were cultured in high glucose Dulbecco’s modified Eagle’s medium (DMEM; Life Technologies) containing GlutaMAX™ and supplemented with 10% embryonic stem cell-qualified fetal bovine serum (ES-FBS; Invitrogen, Carlsbad, CA) and 1% penicillin/streptomycin.

#### Human Umbilical Cord Mesenchymal Stem Cells (UC-MSCs)

Whole umbilical cords collected from consenting mothers with healthy full-term pregnancies were provided by the Anthony Nolan Cell Therapy Centre under ethical approval. Information on the ethical approval held by the Centre, that acts as a research tissue bank, can be found at: http://www.anthonynolan.org/about-us/accreditation-and-regulation. UC-MSCs were isolated from the Wharton’s jelly of the umbilical cord using a modified protocol described by Weiss *et al.*^43^ In brief, the cord was rinsed several times with phosphate buffered saline (PBS; PAA Laboratories, Linz, Austria), and the blood vessels removed prior to the cord being cut into small pieces (3–5 mm^3^) and incubated with an enzyme solution (composed of 0.5 mg/mL of hyaluronidase and 0.5 mg/mL of collagenase) in a MACS MixTM Tube Rotator (Miltenyi Biotec) at 37 °C. After 1 hour, 0.125% trypsin was added to the tissue and it was incubated on the tube rotator at 37 °C for an additional 30 minutes. The final product was passed through 70 μm cell strainer (BD Bioscience), washed with PBS, and centrifuged. The pellet was treated with ammonium chloride solution (STEMCELL Technologies) to lyse any remaining red blood cells. The remaining washed cells were centrifuged and resuspended in culture media. Unless stated otherwise, UC-MSCs were cultured in high glucose DMEM containing GlutaMAX™ and supplemented with 10% embryonic stem cell-qualified fetal bovine serum and 1% penicillin/streptomycin. UC-MSCs were also cultured in human umbilical cord blood low enriched plasma, (CP), supplemented with EGF and FGF2; these cells are referred to as UC-MSC (CP) within the text. CP was prepared following a protocol modified from Chieregato *et al.*^44^. Briefly, CP was thawed at 37 °C to promote cell disruption. After thawing, CP was heat-inactivated at 56 °C for 30 minutes, centrifuged at 1000 g for 10 minutes at room temperature, and the CP supernatant stored at −20 °C until use.

#### Neural Differentiation

The following neuronal differentiation protocols were used:

Protocol 1. hNSCs were plated in expansion medium on Matrigel coated plates. After 10 days in culture, EGF was withdrawn to induce differentiation. After another 7 days in culture the FGF2 and heparin were also withdrawn from the medium and the cells were maintained in DMEM/F12 medium with Glutamax, supplemented with 1% penicillin/streptomycin, 1% N2 supplement, 2% B27 supplement, 50 μg/ml BSA fraction V, 10 ng/ml human recombinant nerve growth factor (βNGF) and 10 ng/ml human recombinant brain-derived nerve growth factor (BDNF; both Peprotech) for another 7 days to allow maturation.

Protocol 2. hNSCs were plated in expansion media containing laminin and after reaching confluence differentiation was induced using DMEM/F12-Glutamax containing 1% penicillin/streptomycin, 10 μM forskolin, 5 mM KCl, 2 mM valproic acid (VPA), 1 μM hydrocortisone and 5 μg/ml insulin. After 10 days the medium was changed to Neurobasal A medium supplemented with 1% L-Glutamine, 1% penicillin/streptomycin and 2% B27 supplement for another 20 days to allow maturation. Differentiated cells were either collected for analysis of gene expression or fixed in 4% paraformaldehyde and characterized by immunocytochemistry as described below.

Protocol 3. A modified “Protocol 2” was used to assess neural differentiation potential of ADSCs and UC-MSCs. When cells became confluent the medium was changed to DMEM containing Glutamax, 1% penicillin/streptomycin, 10% ES-FBS, 10 μM forskolin, 5 mM KCl, 2 mM VPA, 1 μm hydrocortisone and 5 μg/ml insulin. After 3 week differentiation, cells were analyzed for gene expression. In some experiments, hNSC differentiation was induced with this protocol but ES-FBS was omitted to avoid astrocytic differentiation.

#### Adipogenic Differentiation

Adipogenic differentiation was induced in confluent cells by the addition of a medium (DMEM containing Glutamax, 1% penicillin/streptomycin, 10% ES-FBS, 1 μM dexamethasone, 10 ng/ml insulin, 500 mM 3-isobutyl-1-methylxanthine and 1 mM rosiglitazone). After 3 weeks, cells were fixed in 4% and analyzed semi-quantitatively as previously described^17^.

#### Osteogenic Differentiation

Osteogenic differentiation was induced in confluent cells by the addition of a medium (DMEM containing Glutamax, 1% penicillin/streptomycin, 10% ES-FBS, 0.1 μM dexamethasone, 100 μg/ml ascorbate and 10 mM β-glycerophosphate). After 3 weeks, cells were fixed in ice-cold 70% ethanol and analyzed semi-quantitatively as previously described^17^ or analyzed for gene expression.

#### Chondrogenic Differentiation

Chondrogenic differentiation was induced in confluent cells by the addition of DMEM containing Glutamax, 1% penicillin/streptomycin, 10% ES-FBS, 0.1 μM dexamethasone, 50 μg/ml ascorbate, 10 ng/ml transforming growth factor β1 (TGF β1) and insulin, transferrin and selenium (ITS, Sigma). After 3 weeks, cells were fixed in 4% PFA and analyzed semi-quantitatively as previously described^17^ or analyzed for gene expression.

#### Treatments

Cells were stimulated for 24–48 hours with interferon gamma (IFNγ, Peprotech) at different concentrations (10, 50, 200 ng/ml) as indicated in the results. In some experiments cells were exposed to hNSC conditioned medium for 48 hours.

### Detection of surface antigens by flow cytometry

For cell surface protein analysis, cells were pre-incubated in 2.5% FBS in PBS to prevent any non-specific protein binding for 5 minutes, incubated with antibodies (Supplementary Table S2) diluted in 2.5% FBS in PBS at 4 °C for 10 minutes, and then washed twice with FACS washing buffer. For negative controls cells were stained using the isotype controls. A BD FACSCalibur TM cell analyzer was used to carry out flow cytometry analysis and data were analyzed using FlowJo 6.4.7 software.

### Immunofluorescence

All antibodies used are listed in Supplementary Table S3. Cells or tissues were fixed in 4% PFA prior to immunocytochemical protein detection. After incubation with a blocking/permeabilizing buffer (10% FBS, 3% BSA, and 0.2% Triton-X100 in PBS), cells were incubated with primary antibodies for 2 hours at room temperature and then for 1 hour with secondary antibodies and Hoechst 33258 (2 μg/ml) at room temperature. When double-staining for MHC-II and SOX2, cells were first treated with MHC-II primary antibody, then permeabilized and incubated with the SOX2 antibody. Negative controls were incubated with the secondary antibody only. For live cell staining, cells were washed in ice-cold PBS, blocked using 2.5% FBS in PBS on ice, incubated with the pan-antibody to MHC-II for 15 minutes, extensively washed in PBS, and fixed with 4% PFA. Nuclei were counterstained with Hoechst 33258. Images were acquired either using an inverted microscope Olympus IX71 equipped ORCA-R2 digital camera (Hamamatsu Corp., Bridgewater, NJ) or by confocal laser scanning microscopy (LSM 710, Carl Zeiss, Jena, Germany). Image analysis was performed using Fiji/ImageJ software^45^. Quantification of immunolabeled cells was performed by counting double or triple positive cells in at least 5–6 alternate sections per tissue sample from 5 different embryos.

### Reverse Transcription-Polymerase Chain Reaction (RT-PCR) and Quantitative Real-Time Polymerase Chain Reaction (RT-qPCR)

RNA was extracted from cells and tissue using Tri-Reagent (Life Technologies) according to the manufacturer’s protocol. RNA was retro-transcribed with Moloney murine leukaemia virus reverse transciptase (Promega). mRNA was quantified by real-time quantitative polymerase chain reaction with the 7500 sequence detection system (Applied Biosysytems) and the Quantitect SYBR Green PCR kit (Qiagen) following the manufacturer’s instructions. Primers used are listed in Supplementary Table S3. Gene expression data were normalized using GAPDH housekeeping gene as a reference.

### T cell purification and co-culture

All blood donors gave oral and written consent and cell separation from blood samples was covered by ethical approval 05/Q0603/34 (East London and The City Research Ethics Committee 1). Blood donors were 20- to 35-year-old healthy men and women who were tested to be negative for HIV, hepatitis B virus, and hepatitis C virus. Further exclusion criteria were manifest infections during the last 4 weeks, fever, symptomatic allergies, abnormal blood cell counts, increased liver enzymes, or medication of any kind. Blood from 3 different donors (2 male and 1 female) was used, with one of the male volunteers donating blood two times. Hence independent co-culture experiments were repeated 4 times.

Human lymphocytes were isolated using double-gradient density centrifugation as previously described^30^. The fresh venous blood collected was immediately transferred to a tube containing 3.2% sodium citrate (1:10 dilution). Cells were then separated using the density gradient method: 3 ml of Ficoll Histopaque-10771 was layered on top of 3 ml of Ficoll Histopaque-11911 (both from Sigma) to create discrete layers and 6 ml of blood (diluted 1:1 with RPMI medium) layered on top of the Histopaques. After centrifugation at 400 g at room temperature for 30 minutes, the peripheral blood mononuclear cells (PBMCs) were aspirated from their appropriate layer using a sterile Pasteur pipette and washed with RPMI three times. Cells were resuspended in complete RPMI medium and incubated at 37 °C for 2 hours to remove monocyte macrophages. Post-adherent T lymphocytes (>98% pure) were diluted to 1 × 10^6^ cells/ml in standard hNSC medium and incubated for 10 minutes at 37 °C prior to seeding over stem cell monolayers. For time-lapse recording, about 5 × 10^4^ hNSCs or 2.5 × 10^4^ ADSCs per well were seeded in 12 well tissue culture plates to obtain semi-confluent monolayers. A total of 5 × 10^5^ lymphocytes were seeded onto hNSC/ADSCs monolayers and their movement and interaction were recorded using an inverted microscope (Zeiss Axiovert) equipped with an incubation chamber with temperature and CO_2_ percentage control, and Volocity (Openlab 3.1.5) software. Time-lapse images were taken every 10 seconds for 10 to 30 minutes as specified in the movie legends. Movies were generated using Fiji software. At least 5 hNSC/T cell pairs were tracked for each experimental condition. Four independent experiments with combinations of 2 donors and 2 cell lines were analysed by counting the number of hNSC that had shrunk in the 30 minute co-culture frames and was expressed as the percentage of the total number of hNSC present in the field of view.

### Statistical Analysis

Data are presented as mean ± SEM. The statistical analysis was performed using GraphPad Prism version 5.00 for Windows. Statistical significance was evaluated by Student’s t-test or ANOVA followed by a Tukey post-hoc test. A p value equal to or less than 0.05 was considered as statistically significant.

## Additional Information

**How to cite this article**: Vagaska, B. *et al.* MHC-class-II are expressed in a subpopulation of human neural stem cells *in vitro* in an IFNγ–independent fashion and during development. *Sci. Rep.* **6**, 24251; doi: 10.1038/srep24251 (2016).

## Supplementary Material

Supplementary Information

Supplementary Movie 1

Supplementary Movie 2

Supplementary Movie 3

## Figures and Tables

**Table 1:** Fold increase in IFNγ receptor and in HLA-class II transcripts following neuronal differentiation of hNSCs (protocol 1) and osteogenic differentiation of ADSC and UCMSC as compared to undifferentiated controls assessed by RT-qPCR.

Cell type	IFNγ R1	IFNγ R2	HLA-II
hNSC-Cs17	3.75	2.90	34.44
hNSC-Cs23	7.63	2.36	11.03
hNSC-10wk	3.87	2.32	9.68
ADSC-h11	4.35 ± 0.65	1.46 ± 0.23	
ADSC-h13	5.83 ± 2.91	2.99 ± 1.12	1.70 ± 2.63
UCMSC-3	1.87 ± 1.18	1.68 ± 0.65	
UCMSC-4	0.39 ± 0.13	1.17 ± 0.18	0.25 ± 0.11

Data presented as mean for hNSCs (n = 2, biological replicates per cell line), and mean ± S.E.M. for ADSC/UCMSC (n = 3, biological replicates).
