# Supplementary material for: MHC-class-II are expressed in a subpopulation of human neural stem cells in vitro in an IFNγ–independent fashion and during development
Source: Sci Rep. 2016 Apr 15;6:24251. doi: 10.1038/srep24251 (PMC4832187; doi:10.1038/srep24251)
Supplement: Supplementary Information [file srep24251-s1.pdf]

MHC-class-II are expressed in a subpopulation of human neural stem cells *in vitro* in an IFNγ-independent fashion and during development

[1]Vagaska B, [1]New S.E.P, [2]Alvarez-Gonzalez C, [3]D'Acquisto F, [4]Gomez S.G, [5]Bulstrode N.W, [2]Madrigal A, [1]Ferretti P*

[1] Stem Cells and Regenerative Medicine, UCL Institute of Child Health, London WC1N 1EH, UK, [2]Anthony Nolan Research Institute, London NW3 2QG, UK, and UCL Cancer Institute, [3]William Harvey Research Institute, Barts and The London School of Medicine, Queen Mary University of London, Charterhouse Square, London EC1M 6BQ, UK, [4]Anthony Nolan Cell Therapy Centre, Nottingham NG11 8NS, UK, [5]Department of Plastic Surgery, Great Ormond St. Hospital for Children NHS Trust, London WC1N 3JH, UK

Movie – Legends

Supplementary Figures

Supplementary Tables

Movies – Legends

Movie 1. Interaction of T cell and hNSCs detected by time-lapse microscopy over 10 minutes. Images were taken every 10s at 5 frames/second.

Movie 2. Interaction of T cell and hNSCs detected by time-lapse microscopy over 30 minutes. Images were taken every 10s at 5 frames/second.

Movie 3. Interaction of T cell and hADSCs detected by time-lapse microscopy over 10 minutes. Images were taken every 10s at 5 frames/second.

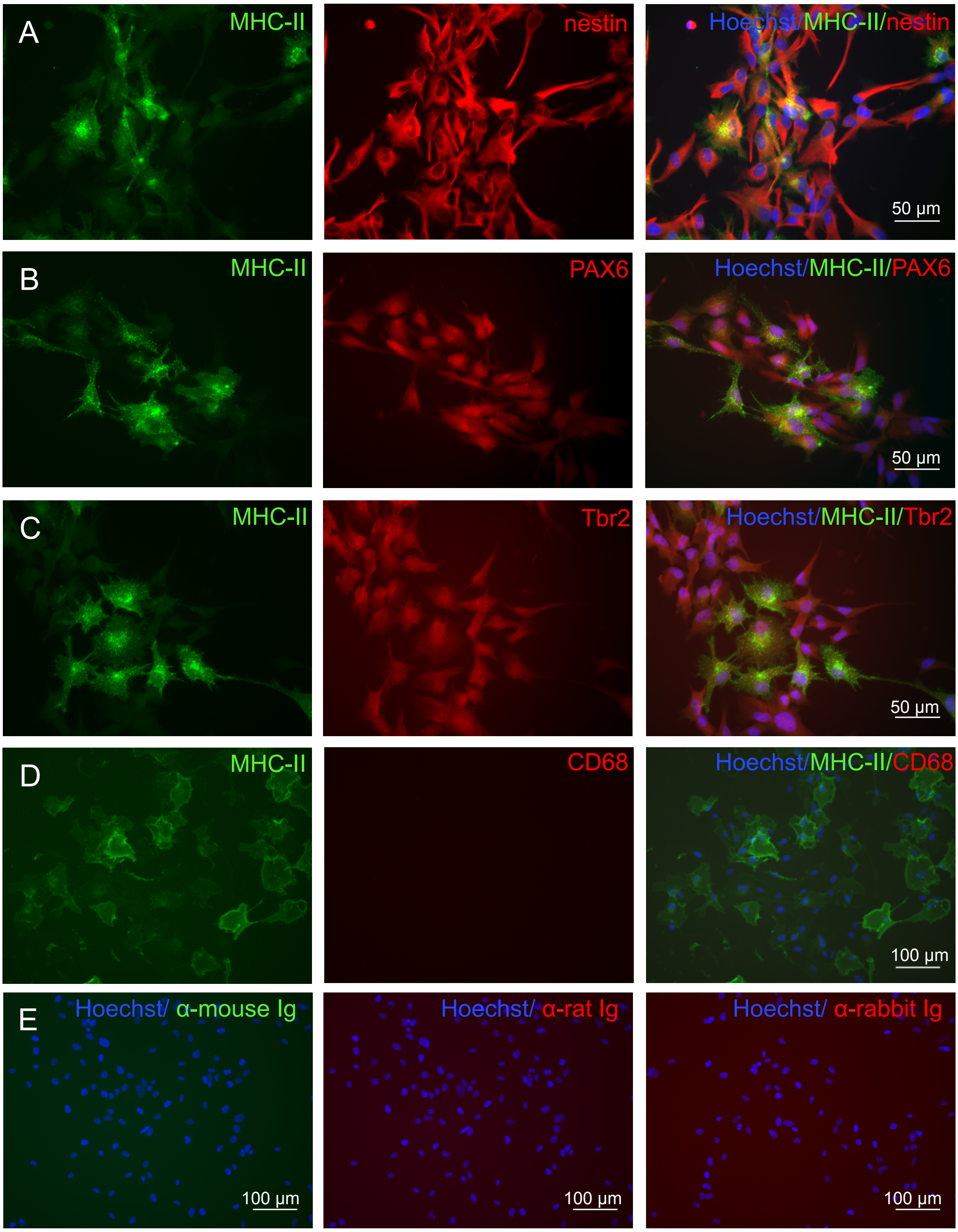

Supplementary Figure S1. Immunofluorescence staining of MHC class II (MHC-II) in combination with microglial and neural stem cell markers in hNSCs *in vitro*. A-C) hNSCs double-labeled for MHC-II and neural stem cell markers: (A) nestin, (B) PAX6 and (C) Tbr2. All cells are positive for the neural stem cell markers with a subset co-expressing MHC-II. Nuclei are counterstained with Hoechst dye (blue). **D)** hNSCs (Cs23) double-labeled for MHC-II and the microglial marker CD68; no CD68-positive cells are observed. **E)** No reactivity is detected in negative controls where primary antibodies are omitted.

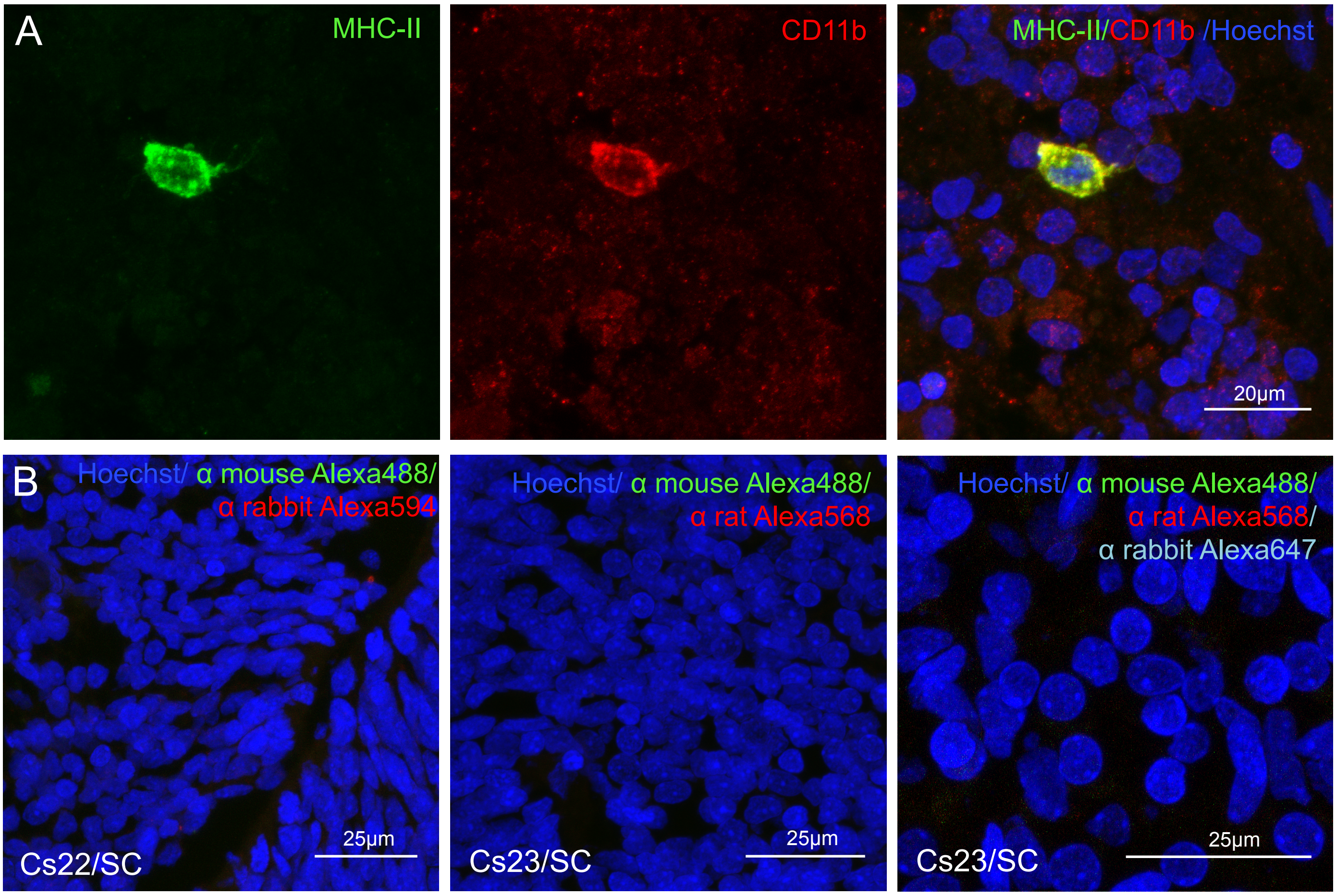

Supplementary Figure S2. Detection of MHC class II (MHC-II) and microglial marker CD11b expression in dorsal root ganglia. Cs22 dorsal root ganglion double-stained for MHC-II and CD11b. Note the double-labelled cell within the ganglion. **B)** Negative controls of immunohistochemical stainings (no primary antibody). Nuclei are counterstained with Hoechst dye (blue).

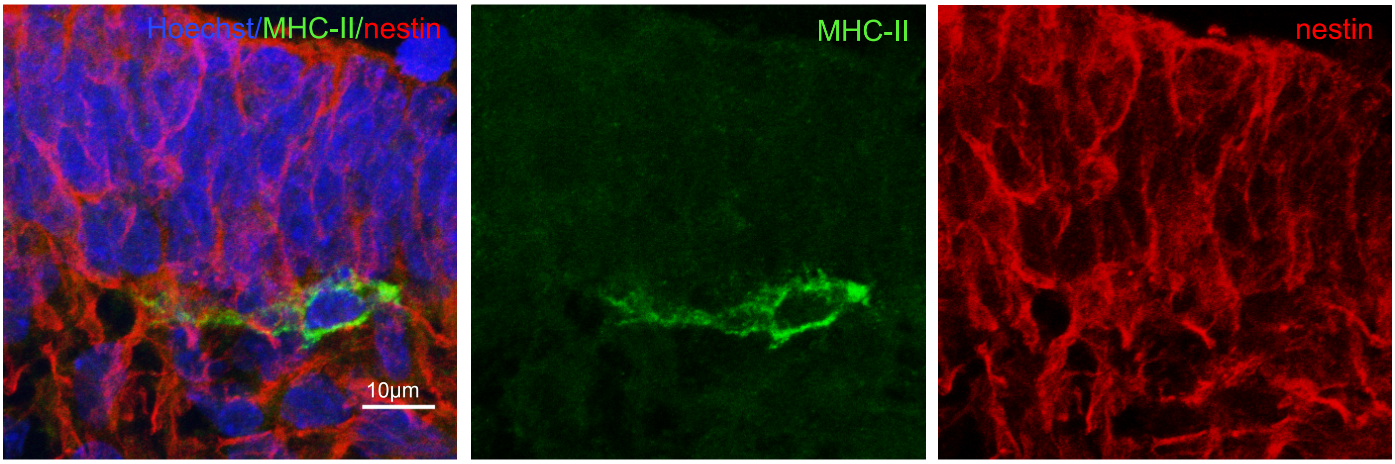

Supplementary Figure S3. Detection of MHC class II and neural marker nestin in human fetal CNS by immunofluorescence. Nestin expression is detected in MHC-II expressing cells adjacent to ventricular germinal layer. Nuclei are counterstained with Hoechst dye (blue).

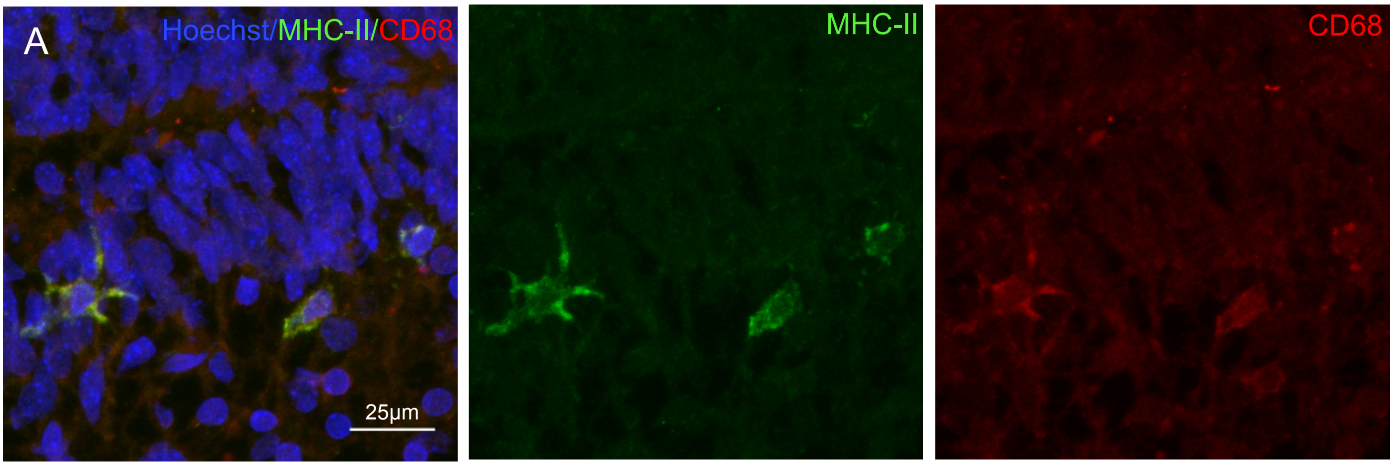

Supplementary Figure S4. MHC class II (MHC-II) and microglial marker CD68 expression in human fetal CNS detected by immunofluorescence. Double-staining for MHC-II and CD68 in spinal cord from embryos at Carnegie stages 22.

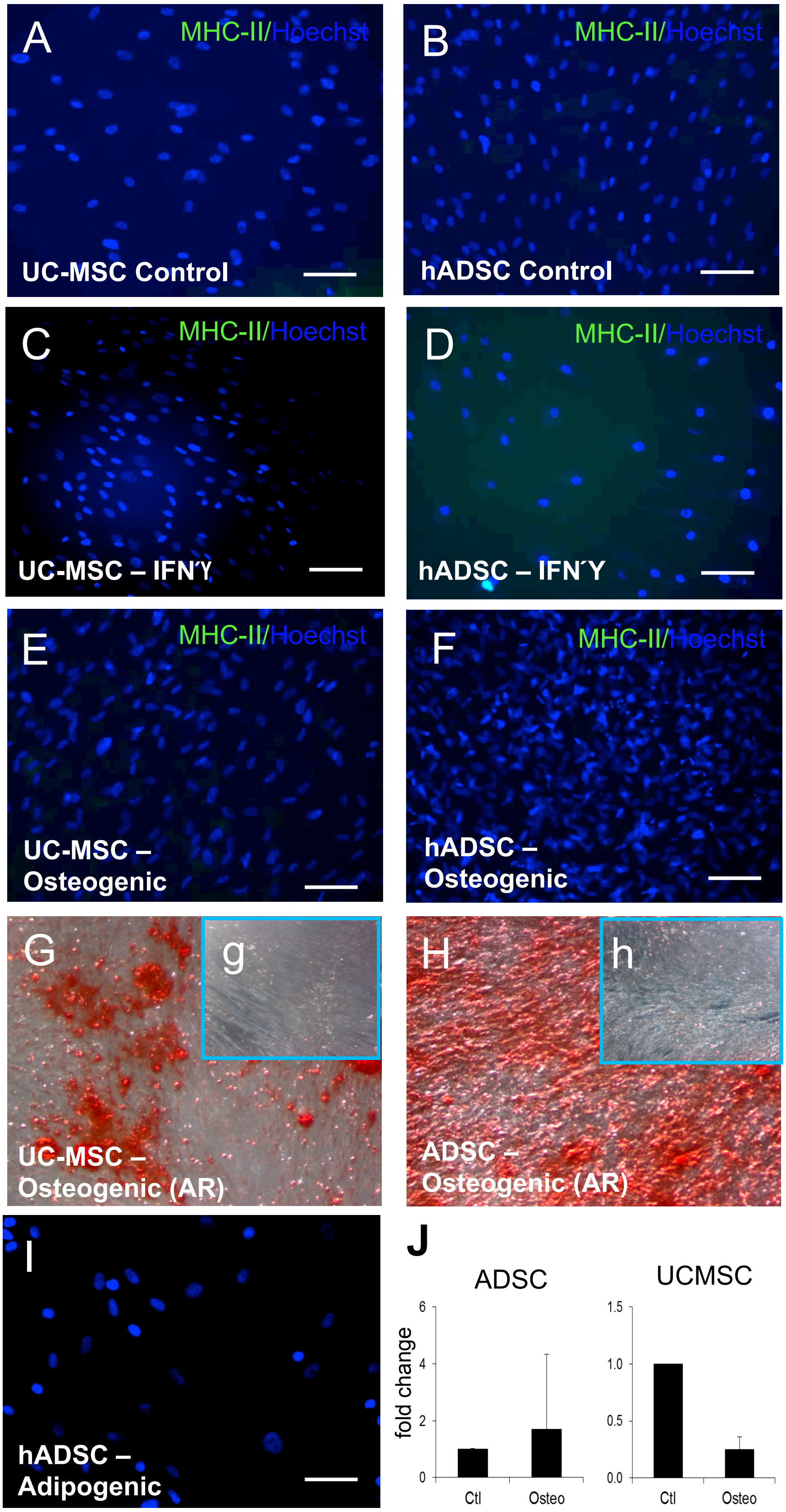

Supplementary Figure S5. Effect of IFNγ stimulation on MHC class II expression in undifferentiated ADSC and UC-MSC lines and following differentiation detected by immunocytochemistry. A-F) Note that all IFNγ treatment does not induce MHC-II expression in either control or differentiated cells. **G-H)** Alizarin red staining demonstrating occurrence of osteogenic differentiation; the insert shows staining in undifferentiated control groups **I)** No MHC-II positive cells are detected following ADSC adipogenic differentiation **J)** Expression of MHC-II in ADSCs and UC-MSCs following osteogenic differentiation detected by RT-qPCR. No increase in HLA-DRA subtype transcripts is observed following osteogenic differentiation in either ADSCs or UC-MSCs, where they appear to have decreased. Scale bars are 100μm.

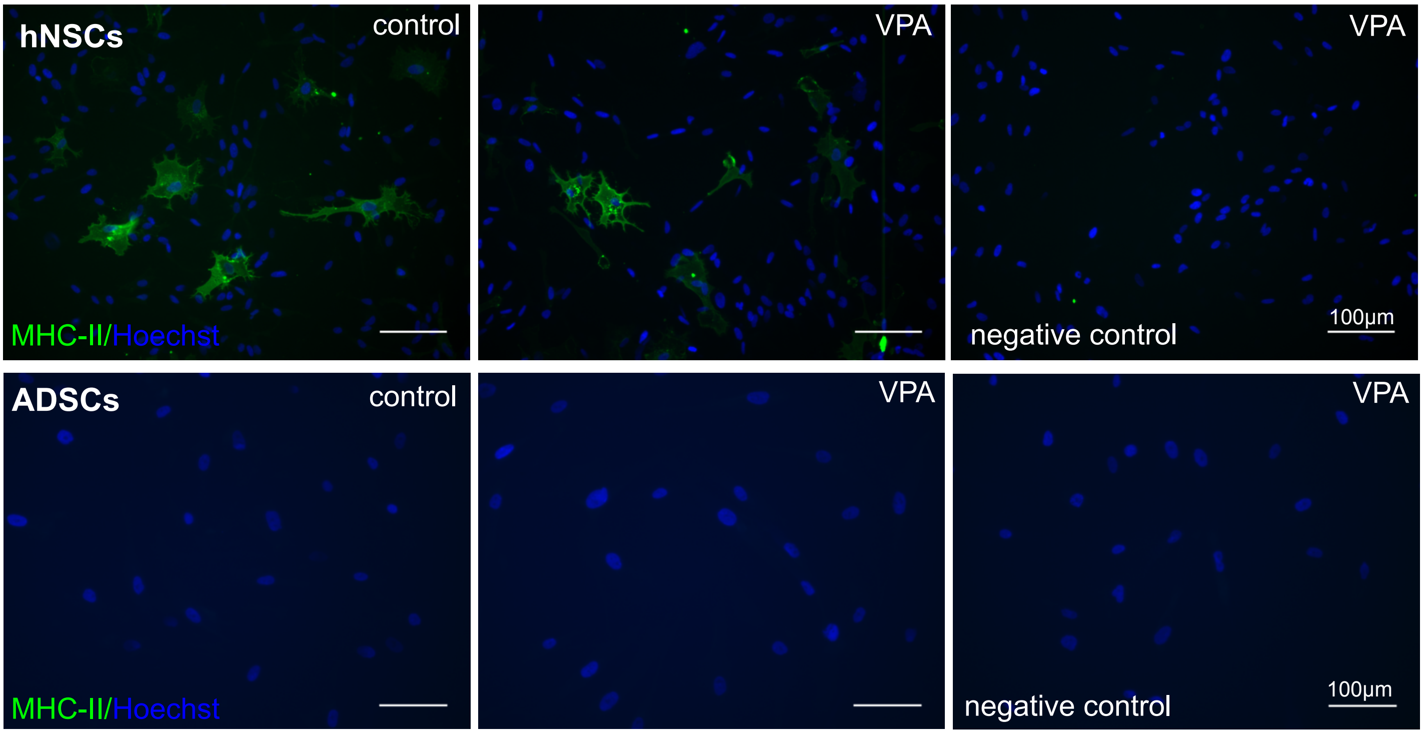

Supplementary Figure S6. Effect of valproic acid (VPA) on MHC-II expression in undifferentiated hNSCs (upper row) and ADSCs (lower row). MHC-II expression assessed by immunofluorescence in control cells and cells treated with 2 mM valproic acid (VPA) for 24 hours (this is the VPA concentration used in neuronal differentiation experiments; a 24 hour treatment with histone deacetylase inhibitors has been shown to be sufficient to induce surface expression of MHC-II in other systems). MHC-II reactivity is clearly detected in hNSCs, but not in ADSCs. VPA treatment has no observable effect on MHC-II expression in either cell type. No staining is present in negative controls (no primary antibody). All images are at the same magnification.

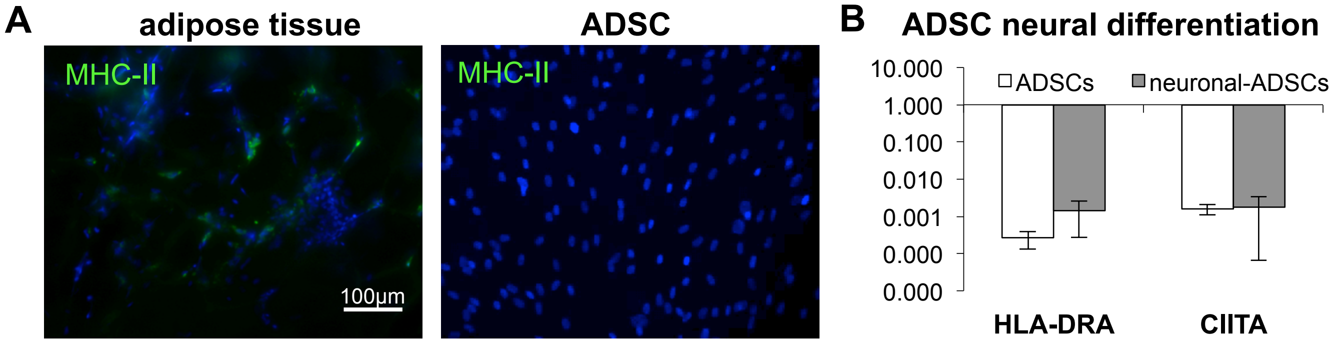

Supplementary Figure S7. MHC class II expression in adipose tissue and ADSCs and following ADSC neuronal differentiation. A) Detection of MHC-II proteins by immunocytochemistry in a section of adipose tissue and cultured ADSCs. A few MHC-II positive cells are present in the fat tissue but not in ADSC cultures. **B)** Detection of MHC-II and CIITA transcripts by RT-qPCR (expression normalized to hNSCs). MHC-II and CIITA mRNA levels are much lower than in hNSCs and do not change following neuronal differentiation induction.

Supplementary Tables

Supplementary Table S1. Quantification of the MHC-II/CD11b and MHC-II/SOX2 double-positive cells and MHC-II/SOX2/CD11b triple-positive cells in the developing human foetal CNS.

	MHC-II+/CD11b+ ¶	SOX2+/MHC-II+	CD11b+ and SOX2+/ MHC-II+
**% of cells**	92.5	87.9	88.2
**SD (%)**	7.09	4.87	5.57
**Total number of counted cells**	88	76	69

Numbers indicate double/ triple positive cells in spinal cords from developing embryos at Carnegie stages 22 and 23 expressed as mean percentage of CD11b-positive ¶ or MHC-II-positive cells (n=5 different embryos).

Supplementary Table S2. Induction of MHC-II by IFNγ and upon differentiation assessed by immunocytochemistry.

Cell type	control	IFNγ treated
hNSC		
undifferentiated	(√)	√
neuronal differentiation	√	ND
astrocytic differentiation	-	√
ADSC		
undifferentiated	-	-
osteogenic differentiation	-	-
adipogenic differentiation	-	-
neuronal differentiation	-	-
UC-MSC		
undifferentiated	-	-
osteogenic differentiation	-	-
Chondroblasts		
undifferentiated	-	-
chondrogenic differentiation	-	-

ND: not done, as MHC-class II expression is high in differentiated cells.

Supplementary Table S3. Primary and secondary antibodies used in this study for immunohistochemistry / immunocytochemistry.

Antibody	Species	Types	Dilution	Source
**Primary**				
HLA-DR, -DP, -DQ-FITC	Mouse	IgG2a	1:5	BD Pharmingen
HLA-A, -B. -C-PE	Mouse	IgG1	1:5	BD Pharmingen
IgG1, k Isotype -FITC Control	Mouse	IgG1, k	1:5	BD Pharmingen
IgG1, k Isotype-PE Control	Mouse	IgG1, k	1:5	BD Pharmingen
IgG2a, k Isotype-FITC Control	Mouse	IgG2a, k	1:5	BD Pharmingen
MAP2	Mouse	IgG	1:100	Life Technologies
GFAP	Rabbit	IgG	1:1000	Millipore
SOX2	Rabbit	IgG	1:200	Millipore
Doublecortin	Rabbit	IgG	1:200	Cell Signaling
Nestin	Rabbit	IgG	1:500	Millipore
PAX6	Rabbit	IgG	1:100	BioLegend
Tbr2	Rabbit	IgG	1:500	Abcam
CD68	Rabbit	IgG	1:100	Santa Cruz
CD11b	Rat	IgG	1:100	Abcam
CD45	Mouse	IgG1	1:15	BD Pharmingen
CD4	Mouse	IgG1	1:15	BD Pharmingen
CD8	Mouse	IgG1	1:15	BD Pharmingen
**Secondary**				
anti-mouse IgG Alexa Fluor® 488	Donkey	IgG	1:400	Molecular probe, Invitrogen
anti-rabbit IgG Alexa Fluor® 594	Donkey	IgG	1:400	Molecular probe, Invitrogen
anti-rabbit IgG Alexa Fluor® 647	Goat	IgG	1:400	Molecular probe, Invitrogen
anti-rat IgG Alexa Fluor® 568	Goat	IgG	1:400	Molecular probe, Invitrogen

All antibodies were diluted in the blocking/permeabilising buffer (10% FBS, 3% BSA, and 0.5% Triton-X100 in PBS) and cells extensively washed with PBS after antibody incubation.

Supplementary Table S4. Primers used for RT-qPCR

Gene	Primers (5'-3')
GAPDH	For TGATGACATCAAGAAGGTGGTGAAG<br>Rev TCCTTGGAGGCCATGTGGGCCAT
HLA -DRA	For AGACAAGTTCACCCCACCAG<br>Rev AGCATCAAACTCCCAGTGCT
CIITA	For CCGACACAGACACCATCAAC<br>Rev TTTTCTGCCCAACTTCTGCT
IFNγ receptor 1	For TGGAATCGCTAACTGGCACT<br>Rev TGCTGTATGCCGAGATGGAAA
IFNγ receptor 2	For GTAAATGGTTCACGGCCGAC<br>Rev CCCGACAGTCACATTCCGA
IFNγ	For TGACCAGAGCATCCAAAAGA<br>Rev CTCTTCGACCTCGAAACAGC
